# Biexcitons in monolayer transition metal dichalcogenides tuned by magnetic fields

**DOI:** 10.1038/s41467-018-05643-1

**Published:** 2018-09-13

**Authors:** Christopher. E. Stevens, Jagannath Paul, Timothy Cox, Prasana K. Sahoo, Humberto R. Gutiérrez, Volodymyr Turkowski, Dimitry Semenov, Steven A. McGill, Myron D. Kapetanakis, Ilias E. Perakis, David J. Hilton, Denis Karaiskaj

**Affiliations:** 1 Department of Physics, University of South Florida, 4202 East Fowler Ave., Tampa, FL 33620 USA; 2 Department of Physics, University of Central Florida, Orlando, FL 32816 USA; 3 National High Magnetic Field Laboratory, Florida State University, Tallahassee, FL 30201 USA; 4 Department of Physics, University of Alabama at Birmingham, Birmingham, AL 35294 USA

## Abstract

We present time-integrated four-wave mixing measurements on monolayer MoSe_2_ in magnetic fields up to 25 T. The experimental data together with time-dependent density function theory calculations provide interesting insights into the biexciton formation and dynamics. In the presence of magnetic fields the coherence at negative and positive time delays is dominated by intervalley biexcitons. We demonstrate that magnetic fields can serve as a control to enhance the biexciton formation and help search for more exotic states of matter, including the creation of multiple exciton complexes and excitonic condensates.

## Abstract (web summary)

Biexciton complexes in atomically thin transition metal dichalcogenides have unusually large binding energies. Here, the authors explore biexciton formation dynamics in monolayer MoSe_2_ in the presence of magnetic fields up to 25 T.

## Introduction

Monolayer two-dimensional transition metal dichalcogenide (TMDs) have generated excitement because of their interesting fundamental physical properties and promising applications on electronic and optoelectronic devices^1–13^. The strong unscreened Coulomb interactions and the anisotropic dielectric environment in these materials lead to the formation of strongly bound excitons, trions, and biexcitons^14–18^. Biexcitons in monolayer TMDs have unusually large binding energies and have been of interest for both fundamental studies and device applications^19^. Biexcitons have been suggested as building blocks for quantum logic gates, biexciton lasing devices, and entangled photon sources^20–23^. From a fundamental physics perspective biexcitons are also an important example where the mean field Hartree–Fock theory fails and higher-order electronic correlations have to be included^24–28^. Furthermore, in magnetic fields the excitons in TMDs experience valley Zeeman splitting, which can be used to magnetically tune the polarization and coherence of the excitonic valley pseudospin^29–33^.

**Fig. 1:**
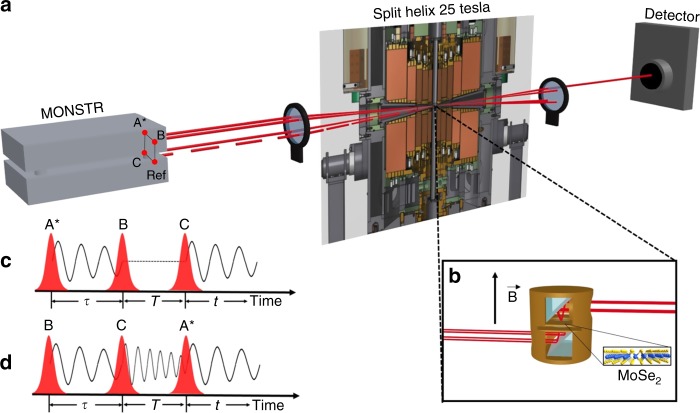
Experiment. **a** Schematic of the experimental setup: The three laser beams are provided by the multidimensional optical nonlinear spectrometer (MONSTR)^57^. Three beams labeled as A^*^, B, and C are used to generate the FWM signal, where A^*^ corresponds to the phase conjugate beam. The beams are aligned in the three corners of a square. The FWM signal generated at the sample propagates along the missing corner (direction −${\mathbf{k}} _{\mathrm{a}} + {\mathbf{k}} _{\mathrm{b}} + {\mathbf{k}} _{\mathrm{c}}$) and is collected by the detector. The samples are kept at 10 K inside the magneto-optical cryostat. **b** The magnetic fields up to 25 T are applied perpendicular to the sample surface in Faraday geometry. **c** Pulse sequence leading to positive delay FWM signal. **d** When the phase conjugate pulse A^*^ arrives last, the negative delay signal is generated due to multiple exciton correlations

In semiconductor materials, excitons correspond to two-particle correlations in the lowest order approximation, whereas biexcitons are four-particle correlations^24^. In linear photoluminescence spectroscopy it is difficult to distinguish between contributions originating from biexcitons and other sources resulting in a nonlinear increase of the peak intensity with excitation density. Coherent nonlinear spectroscopy can separate these different contributions. Two-dimensional Fourier-transform spectroscopy based on the four-wave mixing (FWM) signal is an excellent example where the two and four particle correlations generate different features in the two-dimensional frequency spectra^19,34–36^. Alternatively, time-integrated FWM spectroscopy, combined with microscopic theoretical calculations can already provide important insights. In a time-integrated FWM experiment the three pulses A^*^, B, and C are sent to the sample separated by the time delays *τ* and *T* (Fig. 1c, d). The pulse sequence is varied from negative delays, where the conjugate pulse A^*^ trails behind B and C to positive delay where A^*^ arrives ahead of B and C. Both the negative and positive delay signals have contributions from biexcitons, however the negative delay response originates purely from four-particle correlations^37,38^.

The time-integrated FWM signal in the negative time delay regime is the most striking signature of the difference between nonlinear optical response of a collection of atoms and a solid state. This seemingly counterintuitive behavior that appears to violate causality is a result of the Coulomb interactions, which are dominant in semiconductors. Two-dimensional materials, such as TMDs are excellent test environments for such effects due to the very strong unscreened Coulomb interactions and the anisotropic dielectric environment. In the absence of magnetic fields, the existence of the four-particle exciton–exciton interactions introduces the negative time delay decay on the time-integrated FWM signal. Such behavior, described as non-Markovian, has been observed in InGaAs and GaAs quantum wells, where the decay time for the negative delays is twice as fast as for positive delays.

In bulk GaAs the presence of high magnetic fields results in a more complex and interesting behavior. The negative delay signal has been shown to increase very rapidly with increasing magnetic fields at low temperatures, eventually decaying at the same rate as the positive delay signal, instead of twice as fast. Furthermore, the negative delay displays a nonexponential decay indicating non-Markovian memory effects^39,40^. The underlying cause for this effect is that the excitons created in the semiconductor by one laser pulse are strongly distorted by the magnetic field. Therefore, they acquire a quadrupole moment and through it they generate a coherent four-particle correlation in the medium. It corresponds to a two-photon active coherence and cannot deliver one photon emission by itself. Thus it is stored in the medium until the second pulse triggers that emission. This “coherent memory” can be interpreted as a non-Markovian process involving the two-particle correlation polarization waves interacting with a bath of four-particle correlations^24^. In bulk GaAs the non-Markovian distorsion is attributed to the degree of freedom excitons have to scatter along the magnetic field axis. The magnetic fields confine the excitons along the plane perpendicular to the field direction, creating boson like magneto-excitons. This quantization leads to strong exciton–exciton interactions along the magnetic field axis and thus to a strong negative delay signal^41^.

Despite the conventional picture, in monolayer TMDs even in the absence of magnetic fields we observe a negative time delay signal that equals the positive time delay signal. Furthermore, the appearance of the negative delay signal becomes more pronounced when cross-circular polarizations are used in a way that the first pulse excites the **K** valley, whereas the second pulse excites the **K′** valley, leading to the formation of intervalley biexcitons^19^. The effect of cross-circular polarizations is due to the more preferable formation of biexcitons between opposite spin excitons. The magnetic field further tunes these interactions leading to an increase of the dephasing time. Time-dependent density-functional theory (DFT) calculations, in their density-matrix representation can microscopically track the different contributions to the FWM signal and provide very interesting insights into the effect of the external magnetic fields on the quasiparticle ordering^34,35^. These calculations reveal that in magnetic fields, the experimentally observed dephasing process is dominated by biexcitons for both positive and negative delays. This indicates an interesting ordering of the electrons and holes by means of strong Coulomb interactions into a four-particle correlated state. The optical dephasing takes place fully in a four-particle ordered state comprised of intervalley biexcitons, creating favorable conditions for multiexciton superfluidity. This system offers new opportunities for creating exciton condensates^42^, developing ultrathin biexciton lasers, and generating polarization-entangled photon sources^21,23,43,44^.

## Results

### Linear polarization measurements

**Fig. 2:**
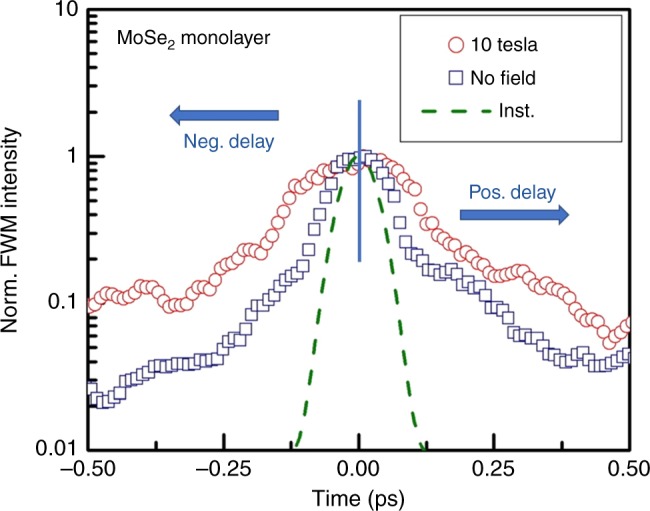
Time-integrated FWM using linear polarizations. Time integrated FWM intensity as a function of the time delay at zero field (blue squares) and 10 T (red circles) using linear horizontal polarizations. The dashed green curve corresponds to the instrumental response. The positive time delay corresponds to the pulse sequence shown in Fig. 1c, whereas negative time delay corresponds to the pulse sequence shown in Fig. 1d. The phase conjugate pulse A^*^ is scanned continuously from negative time delay (arriving at the sample last) to positive time delay (arriving at the sample first). The time delay *T* between pulses B and C is kept fixed at 0 fs

We start by discussing the time-integrated FWM measurements on the monolayer MoSe_2_. The time-integrated FWM signal is plotted as function of the time delay between pulse A^*^ and the pulses B and C is shown Fig. 2. Linear polarizations excite both the **K** and **K**′ valleys equally and are used in order to probe the effect of the magnetic fields independently of the valley spins. We see an increase in the coherence time *T*_2_ to ~600 from ~400 fs when measured at 10 T compared to zero magnetic fields. Such an increase is even more pronounced in the negative delay signal. The excitonic binding energy of ~0.5 eV is energetically large compared to the 10 T external magnetic fields, causing valley Zeman splitting in the order of ~0.22 meV  T^−1^ ^15,29–32,45–47^. Therefore, the excitons should be only slightly perturbed by the magnetic field. Nevertheless, it is the splitting of the electrons and holes into discrete levels by the external magnetic fields that leads to reduced excitonic scattering. This increases the lifetime and suppresses the dephasing process, leading to longer dephasing times.

### Circular polarization measurements

**Fig. 3:**
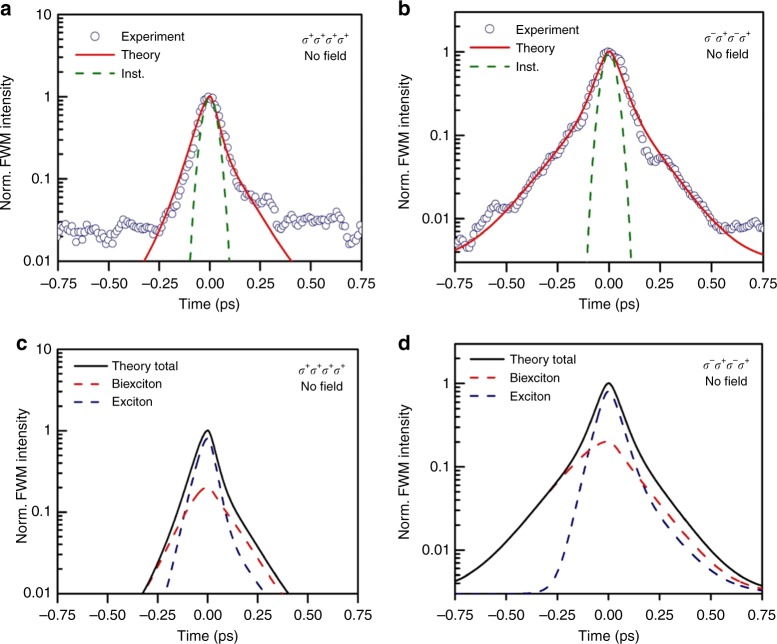
Time-integrated FWM using circular polarizations. Time-integrated FWM intensity as a function of the time delay *τ* at zero magnetic fields for two different polarization sequences (*σ*^+^*σ*^+^*σ*^+^*σ*^+^) (**a**) and (*σ*^−^*σ*^+^*σ*^−^*σ*^+^) (**b**), where the individual polarizations correspond to the laser pulses A^*^, B, C, and detection, respectively. The time delay *T* between pulses B and C is kept fixed at 0 fs. Blue circles are the experimental data, whereas the red lines are the time-integrated FWM calculated using time-dependent DFT. The dashed green lines correspond to the instrumental response. The background noise level is lower for (*σ*^−^*σ*^+^*σ*^−^*σ*^+^) polarizations, due to better rejection of scattered light originating from the excitation laser pulses. **c**, **d** The final calculated time-integrated FWM curves (black line) are plotted together with contributions arising solely from the two-particle exciton correlations (blue dashed line) and four-particle biexciton correlations (red dashed line) for the two polarization sequences

Using left (*σ*^+^) or right (*σ*^−^) handed circularly polarized light at the lowest A exciton resonance energy, excitons are created preferentially in the **K** or **K**′ valleys at the corners of the first Brillouin zone^16,48,49^. We assess in this way the role of the valley spins on the exciton–exciton interactions. We apply two polarization sequences (*σ*^+^*σ*^+^*σ*^+^*σ*^+^) cocircular and (*σ*^−^*σ*^+^*σ*^−^*σ*^+^) cross-circular, where the individual polarizations correspond to the laser pulses A^*^, B, C and detection, respectively^19,50^. The cocircular polarizations lead to exciton formation in only one valley **K**, whereas using the cross-circular polarizations excitons in different valleys **K** and **K**′ are created. The time-integrated FWM data for cocircular and cross-circular polarizations are shown in Fig. 3a, b, respectively. Both the negative and positive decays become much longer for cross-circular polarizations and the dynamics are well reproduced by the time-dependent DFT calculations. The increase of the decay time is attributed to the reduced exciton–exciton scattering due to excitons of different spins in different valleys being excited.

### Theoretical approach

The time-integrated FWM signal is determined by the third order nonlinear polarization using the density-matrix formalism. Here, we employ the time-dependent DFT in its density-matrix representation to develop the equations of motion that describe the polarization dynamics^34,35,51–54^. For realistic simulations of the experiment, we consider a four-band model, including the two top valence and two bottom conduction bands, in order to describe the dynamics of the two valleys at the **K** and **K**′ points of the Brillouin zone. The light-induced electronic excitations taking place at the **K** and **K**′ valleys form excitons due to the Coulomb electron–hole interactions.

The quantities relevant to the time-integrated FWM signal are the polarizations at each of two valleys $\rho _{\bf{K}}^{cv}(t)$ and $\rho _{{\bf{K}}\prime }^{cv}(t)$, where the indexes *c* and *v* run over the two valence and two conduction bands, respectively. Within the framework of the density-matrix time-dependent DFT excitonic effects are accurately described using the screened Slater exchange-correlation potential with the nonadiabatic kernels which display a Coulomb-like long-range behavior and include memory effects^52,55^. The realistic Coulomb interactions enable very clear and specific insight into the nature of the quasi-particles formed. In order to include the biexciton contributions, the equations of motion (Supplementary Note 2 equations B18–19 and B20–21) are expanded up to third order. By switching off the (~*B*) terms in equations (Supplementary Note 2 equations B18–C19), the biexciton effects are ignored and the resulting time-integrated FWM signal originates solely from the excitonic contribution. Therefore, we can assess the role of the exciton and biexciton contributions on the time-integrated FWM signal separately and draw conclusions about their contribution at both, positive and negative time delays.

The total calculated time-integrated FWM has been plotted together with contributions arising only from the exciton correlations and four-particle bound biexciton correlations for each polarization sequence. The total simulated FWM signal containing all contributions reproduces well the experimental data in Fig. 3a, b. At negative delays the FWM signal can originate from four-particle unbound exciton–exciton scattering and bound biexcitons. However, we refer to bound biexcitons when we plot the biexcitonic contributions. In Fig. 3c, d, we separately plot the excitonic and biexcitonic contributions to the total time-integrated FWM signal. The negative delay signal is dominated by the biexciton response, whereas the positive delay signal is almost equally due to both, excitons and biexcitons. At zero magnetic fields, this holds true for both polarization sequences, although for cross-circular polarizations the dephasing time is much longer because of excitons of opposite spin in different valleys being created^19^. The nature and ratio between the two-particle exciton, four-particle exciton–exciton, and bound biexciton interactions have been obtained directly from the time-dependent DFT and are a result of the strength of the Coulomb interactions.

### High magnetic field measurements

**Fig. 4:**
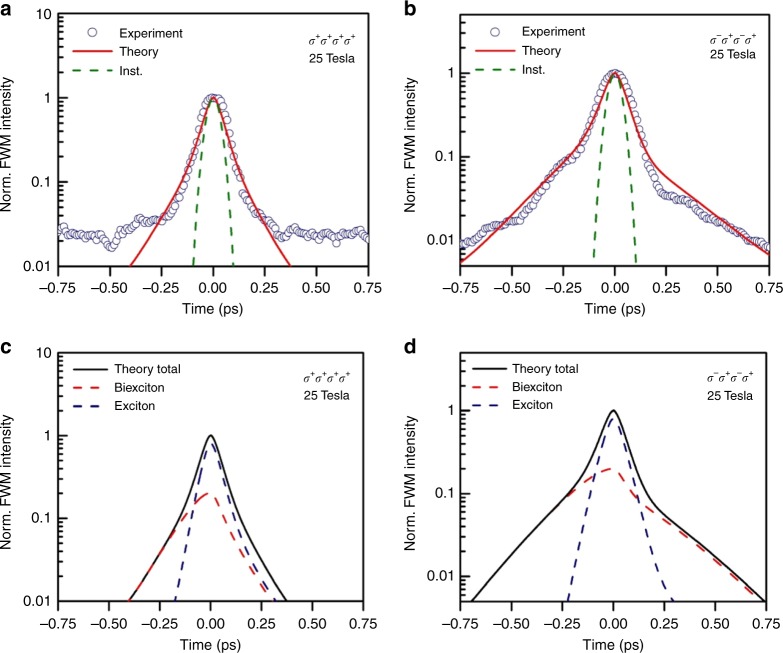
Time-integrated FWM at high magnetic fields. Time-integrated FWM intensity as a function of the time delay *τ* at 25 T for two different polarization sequences (*σ*^+^*σ*^+^*σ*^+^*σ*^+^) (**a**) and (*σ*^−^*σ*^+^*σ*^−^*σ*^+^) (**b**), where the individual polarizations correspond to the laser pulses A^*^, B, C, and detection, respectively. The time delay *T* between pulses B and C is kept fixed at 0 fs. Blue circles are the experimental data, whereas the red lines are the time-integrated FWM calculated using time-dependent DFT. The dashed green lines correspond to the instrumental response. The background noise level is lower for (*σ*^−^*σ*^+^*σ*^−^*σ*^+^) polarizations, due to better rejection of scattered light originating from the excitation laser pulses. **c**, **d** The final calculated time-integrated FWM curves (black line) are plotted together with contributions arising solely from the two-particle exciton correlations (blue dashed line) and four-particle biexciton correlations (red dashed line) for the two polarization sequences

External magnetic fields can lead to the valley Zeeman splitting in monolayer TMDs, which can be tuned by the field, causing a linear shift between the two Zeeman levels of ~0.22 meV  T^−1^ for MoSe_2_^29,31^. Furthermore, it leads to an increase in the degree of circular polarization of the emitted light, by lifting the degeneracy of the two valleys at the **K** and **K**′ points of the Brillouin zone^32^. The discrete nature of the electron and hole energy levels in external magnetic fields also creates favorable conditions for exciton condensation and multiple exciton formation. Applying magnetic fields up to 10 T has given rise to long negative delay signals in bulk GaAs, which are comparable with the positive decay time. Unlike bulk GaAs or quasi two-dimensional system, in truly two-dimensional TMDs the particles are highly confined along the magnetic field direction, perpendicular to the two-dimensional atomic plane. Therefore, quasiparticle scattering along the field direction should be restricted by the quantum confinement. In order to investigate the effect of the external magnetic field on this purely two-dimensional system, we have applied external fields up to 25 T shown in Fig. 4a, b and performed time-integrated FWM measurements.

## Discussion

The negative delay signal persists at high magnetic fields, and similar to the zero-field case, is dominated by biexcitons. Surprisingly, at cross-circular polarization the dephasing at positive delays *τ* is also equally dominated by four-particle bound biexcitons. The time-dependent DFT simulations attribute the effect to two main sources. The increased screening due to the presence of additional carriers (four particles complex) leads to longer radiative lifetimes and slower dephasing for biexcitons. Most importantly, the strong magnetic field leads to discrete energy levels for the particles, thus reducing scattering and facilitating higher multiple exciton correlations. Furthermore, the intervalley biexcitons consisting of two excitons with large difference in crystal momentum are unique higher-order bound states with no direct analog in conventional semiconductors. The formation of increasingly separated and discrete levels with increasing magnetic fields is described in more depth in the Supplementary Information (see Supplementary Fig. 3 and Supplementary Note 1). Thus, at higher magnetic fields the formation of biexcitons condensation with very long coherence times could occur.

In conclusion, we measure time-integrated FWM on monolayer MoSe_2_ at the presence of magnetic fields up to 25 T. We observe an increase in the decay time with increasing magnetic fields due to the reduced scattering leading to less decoherence. At zero magnetic fields the time-integrated signal is symmetrical with the negative delay decay time equaling the positive decay. Significant changes are observed when excitons of opposite spins in different valleys are excited, leading to much longer dephasing times for intervalley biexcitons despite the large differences in crystal momenta. In external magnetic fields, we observe interesting ordering of the electrons and holes by means of strong Coulomb interactions into a four-particle correlated state. The optical dephasing takes place in a four-particle ordered state comprised of intervalley biexcitons, creating favorable conditions for interesting new states of matter, including the creation of multiple exciton complexes, exciton superfluidity, and biexciton condensates^42–44^.

## Methods

### Experiment

The monolayer MoSe_2_ was grown by chemical vapor deposition and transferred on a optically transparent quartz substrate^56^. The three laser beams used to generate the FWM signal are provided by the MONSTR instrument described in ref. ^57^. Pulses B and C are kept fixed whereas pulse A^*^ is scanned from negative to positive time delays. The positive delay signal corresponds to the time ordering of the laser pulses shown in Fig. 1c, whereas the negative delay signal time ordering shown in (d). The nonlinear third order response of the sample leads to the FWM signal, which propagates in the phase matching direction −**k**_1_ + **k**_2_ + **k**_3_ along the missing corner of the box formed by the three excitation laser pulses, as shown in Fig. 1a, and the coherent time-integrated FWM signal is collected by the detector. The monolayer MoSe_2_ sample is held at 10 K inside the resistive 25 T split helix magnet. The magnetic field and laser excitations are applied perpendicular to the sample surface shown in Fig. 1b.

### Data availability

The data that support the findings of this study are available from the corresponding author upon reasonable request.

## Electronic supplementary material

Supplementary Information
