# Supplementary material for: Biexcitons in monolayer transition metal dichalcogenides tuned by magnetic fields
Source: Nat Commun. 2018 Sep 13;9:3720. doi: 10.1038/s41467-018-05643-1 (PMC6137096; doi:10.1038/s41467-018-05643-1)
Supplement: Supplementary file 1 — Supplementary Information [file 41467_2018_5643_MOESM1_ESM.pdf]

Supplementary Information

Biexcitons In Monoatomic Transition Metal Dichalcogenides Tuned by Magnetic Fields

Christopher E. Stevens[1], Jagannath Paul[1], Timothy Cox[1], Prasana Sahoo[1],
Humberto R. Gutierrez[1], Volodymyr Turkowski[2], Dimitry Semenov[3], Steven McGill[3]
Myron D. Kapetanakis[4], Ilias E. Perakis[4], David Hilton[4], and Denis Karaiskaj[1*]

[1]*Department of Physics, University of South Florida, 4202 East Fowler Ave., Tampa, Florida 33620*
[2]*Department of Physics, University of Central Florida, Orlando, Florida 32816, USA*
[3]*National High Magnetic Field Laboratory, Florida State University, Tallahassee, FL 30201*
[4]*Department of Physics, University of Alabama at Birmingham, Birmingham, Alabama 35294, USA*

[*]To whom correspondence should be addressed; E-mail: karaiskaj@usf.edu.

This file includes:

Theory sections in Supplementary Note 1 to Supplementary Note 2
Supplementary Figures 1 to 5
Supplementary Note 3: Sample and experimental details
Supplementary references 1 to 17

Supplementary Notes

Supplementary note 1: Ground State calculations within the DFT method.

Zero-field case. The electronic structure of the monolayer $MoSe_2$ was calculated by static DFT calculations, as implemented in the Quantum Espresso package [1], by using the PBE XC ultra soft potential while taking into account spin-orbit coupling (SOC) and Van de Walls (VdW) corrections and sampling the BZ with **14x14x1** ok k-points mesh. The DFT calculations show that the spin-up and spin–down bands are split by 187.3 meV for the valence and 20.7 meV for the conduction band and display opposite spin ordering while the one-particle optical band gap E_G is about 1.45eV (see Supplementary Figure 1). The results for the gap and for the valence band splitting are in a rather good agreement with experimental data 1.58 eV and 180 meV, respectively [2]. Similarly, the conduction band splitting is also in agreement with experimental estimations ~10 meV [3]**.** The band splitting has the opposite spin ordering in K and K' points. A schematic band-ordering in different valleys is shown in Supplementary Figure 2. Due to the strong spin-orbit coupling, the effects of magnetic field can be treated safely as a perturbation, at fields up to 25T, which induces an additional splitting to the zero-field bands.

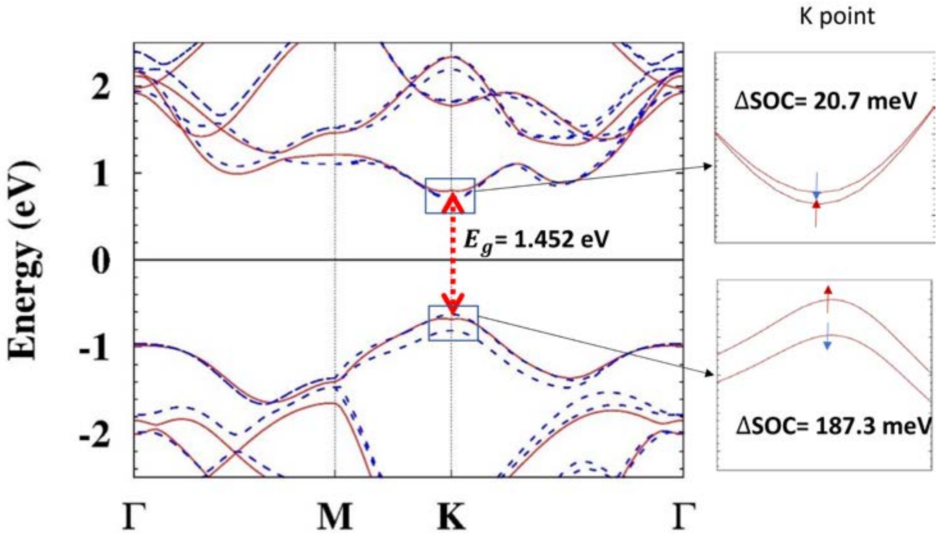

Supplementary Figure 1. Electronic band structure of 1L $MoSe_2$ with SOC-split valence and conduction bands at K-point (at K'-point the order of the split bands is opposite).

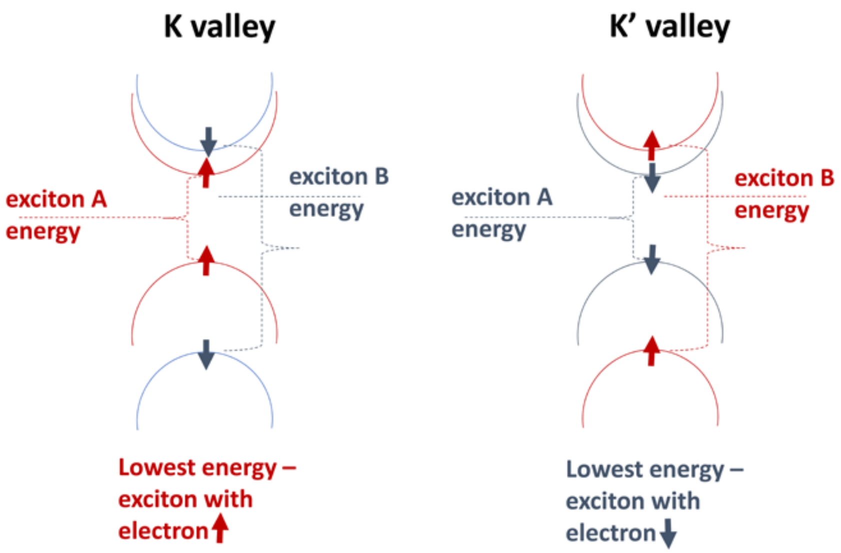

Supplementary Figure 2. Schematic representation of the K and K' valley energetics with marked excitonic levels in 1L MoSe2 at zero magnetic field.

Finite field. At used fields, $B \leq 25T$, the magnetic length $l_B = \sqrt{\frac{\hbar}{eB}} = \frac{25.6}{\sqrt{B(T)}}$ nm is always much larger than the zero-field exciton radius in 1L MoSe2, ~1nm. Also, the cyclotron energy $\hbar\omega_c = \hbar\frac{\sqrt{2}v}{l_B} \sim 0.01\sqrt{B(T)}$eV ($v \sim \frac{at}{\hbar} \sim 10^6\,\frac{m}{s}$ - "Fermi velocity") is much smaller than the exciton binding energy, ~0.5eV, and the band splitting. Therefore, the effects of the magnetic field can be regarded as a perturbation. In the lowest-order approximation, one can regard the DFT (electron) wave functions, as well as exciton and biexciton wave functions and eigenenergies, to be field-independent. However, as it will be shown below, the magnetic field significantly affects the excited charge density, thus one needs to take the Landau level splitting of the electronic and hole spectra into account. Here we briefly show how it is done in practice in this work.

Let us begin by considering an effective low-energy four (two-valence and two-conduction) band model for 1L $MOSe_2$ at zero field. The corresponding minimal Hamiltonian includes the valence $|\phi_v^\tau\rangle = \frac{1}{\sqrt{2}}\left(|d_{x^2-y^2}\rangle + i\tau|d_{xy}\rangle\right)$ ($\tau = \pm1$ is the valley index) and conduction $|\phi_c\rangle = |d_{z^2}\rangle$ bands (see, e.g., Ref. [4]) for 1L MoS2), and has the following form:

$$\hat{H} = at\left(\tau k_x\hat{\sigma}_x + k_y\hat{\sigma}_y\right) + \frac{\Delta}{2}\hat{\sigma}_z - \lambda_v\tau\frac{\hat{\sigma}_z - 1}{2}\hat{s}_z - \lambda_c\tau\frac{\hat{\sigma}_z + 1}{2}\hat{s}_z, \qquad (A.1)$$

where $\hat{\sigma}_z$ is the Pauli matrix operator acting in the valence and conduction band "space", $\hat{s}_z$ is the spin projection matrix; Δ is the bandgap, $2\lambda_v$ and $2\lambda_c$ are splittings of the valence and conduction band, correspondingly.

Diagonalization of this Hamiltonian gives

$$E_{\tau s_z}(k) = \frac{1}{2}(\lambda_v - \lambda_c)\tau s_z \pm \sqrt{a^2t^2k^2 + \frac{1}{4}(\Delta - (\lambda_v + \lambda_c)\tau s_z)^2} \approx \begin{cases} \frac{\Delta}{2} - \lambda_c \tau s_z + \frac{(atk)^2}{\Delta}\ (conduction) \\ -\frac{\Delta}{2} + \lambda_v \tau s_z - \frac{(atk)^2}{\Delta}\ (valence) \end{cases}. \qquad (A.2)$$

In the first line of this equation, "+" corresponds to conduction and "-" to the valence band. Since $\lambda_v \sim 0.09eV, \lambda_c \sim 0.01eV$, t~0.94eV, and ak usually is significantly smaller than π (at not very wide pulses, a small part of the BZ is excited), in the first line all quantities are small compared to the gap Δ~1.47eV, one can get an approximate spectrum in the second line of the equation. Thus the valence band in each valley is split by $2\lambda_v$, but the spin order in K and K' valley is different (for $\tau = 1$ or $\tau = -1$). Similar conclusion is valid for the conduction band.

In the case of magnetic field (for 1L MoS2, see. e.g., Ref. 5), one can make in the Hamiltonian (A.1) the substitution $\vec{k} \to \vec{\pi} = \vec{k} + \frac{e}{c}\vec{A}$, where the field satisfies the Landau gauge $\vec{A}(\vec{r}) = (0, Bx)$, and uses the fact that when the fields are not too strong, $\pi_x + i\pi_y$ coincides with the lowering operator. Thus, in the magnetic case square of the kinetic energy $a^2t^2k^2$ "transforms" into square of the (2D) magnetic energy for given Landau level $\hbar^2\omega_c^2 n$, where $\hbar\omega_c \sim 0.01\sqrt{B(T)}$eV. In other words, parabolic band described by momenta k, transforms into the set of Landau levels that "fill out" the parabola. In this case, one gets the Landau spectrum

$$E_n = \frac{1}{2}(\lambda_v - \lambda_c)\tau s_z \pm \sqrt{\hbar^2\omega_c^2 n + \frac{1}{4}(\Delta - (\lambda_v + \lambda_c)\tau s_z)^2} = \approx \begin{cases} \frac{\Delta}{2} - \lambda_c \tau s_z + \frac{\hbar^2\omega_c^2 n}{\Delta}\ (conduction) \\ -\frac{\Delta}{2} + \lambda_v \tau s_z - \frac{\hbar^2\omega_c^2 n}{\Delta}\ (valence) \end{cases}, \qquad (A.3)$$

where we have used the fact that $\hbar\omega_c \ll \Delta$.

We use the last line approximation to correct the zero-field DFT spectrum in the case of magnetic field. Namely, we use this spectrum to calculate the Slater XC matrix elements that define the exciton-exciton and exciton-biexciton interactions (the number of occupied valence states (within fixed to the zero-field valence band energy range), and hence of the excited-in-the-field states, decreases with the field strength increasing). Notice that, the distance between the Landau levels is small compared to the band splitting. For fields at 25T, for instance, the splitting is approximately 2meV, whereas the conduction band splitting is 20meV, while it increases linearly with respect to the field. The latter affects significantly the number of excited charges (scatterers) at given pulse energy width (see Figure 3) something that plays a crucial role in the field-dependence of the TI-FWM spectrum.

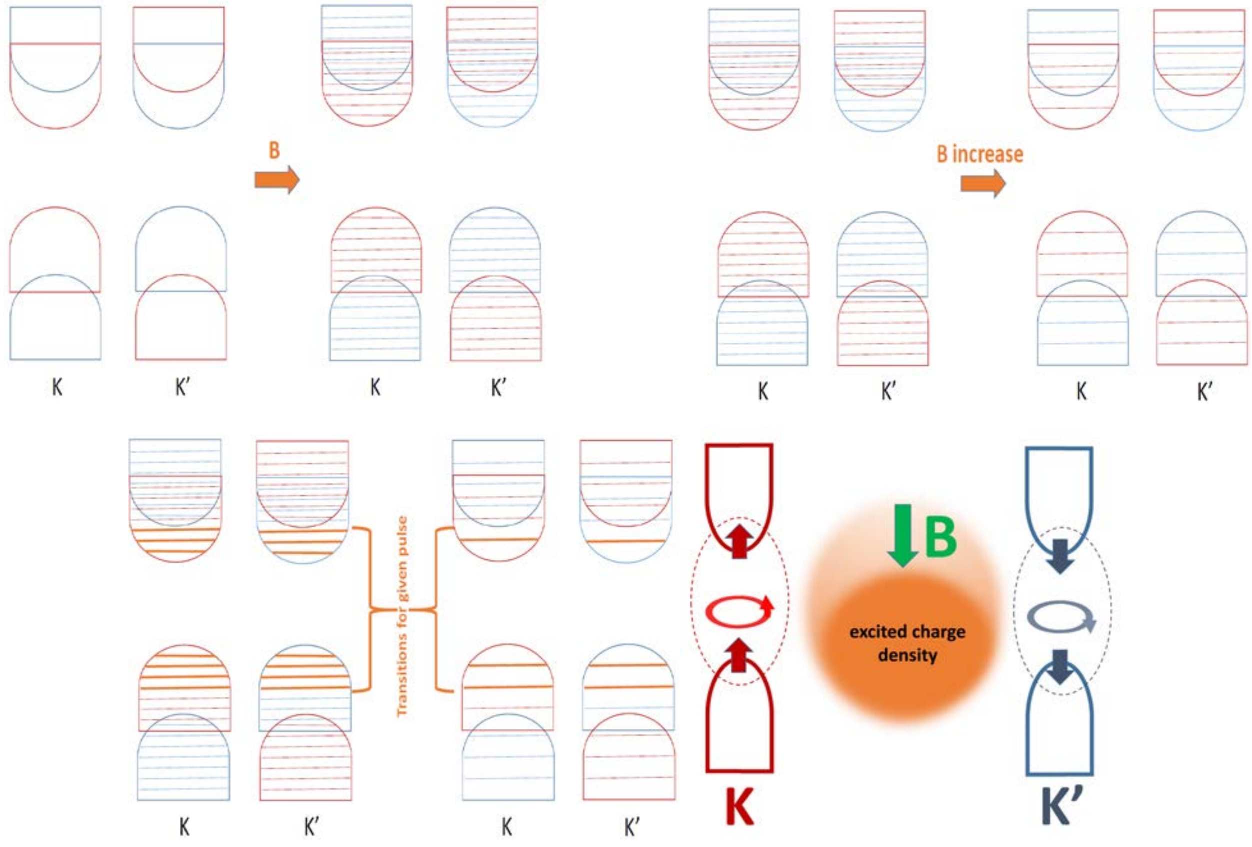

Supplementary Figure 3. Schematic illustration of the effect of the magnetic field on the electronic and absorption spectra of 1L MoSe2. The increased relaxation times in the finite-field case can be explained by reduced screening of the exciton-exciton interaction by free excited carriers as shown at the bottom right figure. The number of such carriers is lower at finite B due to increased distance between the occupied and unoccupied levels as shown in the top two figures. Thus, a pulse of fixed energy width excites less charges as B increases, see bottom left figure.

Supplementary note 2: Third-order polarization equations and TI-FWM signal by TD-DMDFT

To derive the TD-DMDFT equations for excitons, one can start from the TDDFT Kohn-Sham equation

$$i\frac{\partial \Psi_{\mathbf{k}}^{v}(r,t)}{\partial t} = \left[-\frac{\vec{\nabla}^2}{2m} + V_H[n](r,t) + V_{XC}[n](r,t) + e\vec{r}\vec{E}(t)\right]\Psi_{\mathbf{k}}^{v}(r,t), \qquad (B.1)$$

where the terms of the Hamiltonian (inside the brackets) are the kinetic energy operator (first), the Hartree (second) and XC (third) potentials, and the last term in our case is an external homogeneous-in-space electric pulse field; **k** is the wave vector, v are the wave vector and the valence-band index, correspondingly. Equation (B.1) is solved self-consistently with the equation for the electron density

$$n(\mathbf{r},t) = \sum_{l,|\mathbf{k}|<k_F} \left|\Psi_{\mathbf{k}}^{l}(\mathbf{r},t)\right|^2 \qquad (B.2)$$

(*l* is the band index).

To solve Eqs. (B1) and (B2) by using the DM formalism, one expands the KS wave function in terms of the basis (DFT) static wave functions $\psi_{\boldsymbol{k}}^{l}(\boldsymbol{r})$:

$$\Psi_{\mathbf{k}}^{v}(\mathbf{r},t) = \sum_{l} c_{\mathbf{k}}^{l}(t)\psi_{\mathbf{k}}^{l}(\mathbf{r}), \qquad (B.3)$$

where the time-dependent coefficients $c_{k}^{l}(t)$ completely describe the dynamics of the system. To study the system's response, it is more convenient to consider the bilinear combination of c-coefficients, the density matrix:

$$\rho_{kq}^{lm}(t) = c_{k}^{l}(t)c_{q}^{m*}(t) \qquad (B.4)$$

that describes the state occupancies (diagonal elements), and the electron and hole transitions, including the excitonic effects (non-diagonal elements).

The density matrix satisfies the Liouville equation

$$i\frac{\partial \rho_{kq}^{lm}(t)}{\partial t} = [H(t),\rho(t)]_{kq}^{lm}, \qquad (B.5)$$

where the matrix elements of the Hamiltonian are

$$H_{kq}^{lm}(t) = \int \psi_{k}^{l*}(r)H[n](r,t)\psi_{q}^{m}(r)dr. \qquad (B.6)$$

In TD-DMDFT in the case of two different types of excitons (A and A or A and B excitons in K and K' (or 1 and 2) valleys with same or opposite spins), the equations for the third-order polarization have the following form [6]

$$i\frac{\partial}{\partial t}P_{1\boldsymbol{k}}(t) = \left[\varepsilon_{\boldsymbol{k}}^{c_1} - \varepsilon_{k}^{v_1} - \frac{i}{\tau_{X1}}\right]P_{1\boldsymbol{k}}(t) + 2\sum_{q}\int_{-\infty}^{t} dt'\, F_{11\boldsymbol{kq}}(t,t')P_{1\boldsymbol{q}}(t') + \boldsymbol{d}_{\boldsymbol{k}}^{c_1 v_1}\boldsymbol{E}(t)$$

$$+ \sum_{q,p,Q} P_{1q}^{*}(t)\int_{-\infty}^{t} dt'\, H_{11\boldsymbol{kqpQ}}(t,t')P_{1\boldsymbol{p}}(t')\, P_{1\boldsymbol{Q}}(t')$$

$$+ \sum_{q,p,Q} P_{2q}^{*}(t)\int_{-\infty}^{t} dt'\, H_{12\boldsymbol{kqpQ}}(t,t')P_{1\boldsymbol{p}}(t')\, P_{2\boldsymbol{Q}}(t')$$

$$+ \sum\nolimits_{q,p,Q} P_{1q}^{*}(t)\int_{-\infty}^{t} dt'\, F_{21\boldsymbol{kqpQ}}(t,t')\mathrm{B}_{\boldsymbol{12pQpQ}}(t')$$

$$+ \sum\nolimits_{q,p,Q} P_{2q}^{*}(t)\int_{-\infty}^{t} dt'\, F_{22\boldsymbol{kqpQ}}(t,t')\mathrm{B}_{\boldsymbol{12pQpQ}}(t') \qquad (B.7)$$

$$i\frac{\partial}{\partial t}P_{2\boldsymbol{k}}(t) = \left[\varepsilon_{\boldsymbol{k}}^{c_2} - \varepsilon_{k}^{v_2} - \frac{i}{\tau_{X2}}\right]P_{2\boldsymbol{k}}(t) + 2\sum_{q}\int_{-\infty}^{t} dt'\, F_{22\boldsymbol{kq}}(t,t')P_{2\boldsymbol{q}}(t') + \boldsymbol{d}_{\boldsymbol{k}}^{c_2 v_2}\boldsymbol{E}(t)$$

$$+ \sum_{q,p,Q} P_{2q}^{*}(t)\int_{-\infty}^{t} dt'\, H_{22\boldsymbol{kqpQ}}(t,t')P_{2\boldsymbol{p}}(t')\, P_{2\boldsymbol{Q}}(t')$$

$$+ \sum_{q,p,Q} P_{1q}^{*}(t)\int_{-\infty}^{t} dt'\, H_{21\boldsymbol{kqpQ}}(t,t')P_{2\boldsymbol{p}}(t')\, P_{1\boldsymbol{Q}}(t')$$

$$+ \sum_{q,p,Q} P_{2q}^{*}(t)\int_{-\infty}^{t} dt'\, F_{12\boldsymbol{kqpQ}}(t,t')\mathrm{B}_{\boldsymbol{21pQpQ}}(t')$$

$$+ \sum\nolimits_{q,p,Q} P_{1q}^{*}(t)\int_{-\infty}^{t} dt'\, F_{11\boldsymbol{kqpQ}}(t,t')\mathrm{B}_{\boldsymbol{21pQpQ}}(t'). \qquad (B.8)$$

In the above equations,

$$F_{\mathrm{ij}\mathbf{kqk'q'}}(\mathrm{t}-\mathrm{t'}) = \int \mathrm{d}\mathbf{r}_1 \mathrm{d}\mathbf{r}_2 \psi_{\mathbf{k}}^{\mathrm{i}*}(\mathbf{r}_1)\psi_{\mathbf{q}}^{\mathrm{j}}(\mathbf{r}_1)\mathrm{f}_{\mathrm{XC}}(\mathbf{r}_1,\mathbf{r}_2,\mathrm{t}-\mathrm{t'})\psi_{\mathbf{k'}}^{\mathrm{i}}(\mathbf{r}_2)\psi_{\mathbf{q'}}^{\mathrm{j}*}(r_2). \qquad (\mathrm{B}.9)$$

The diagonal elements of F_{ij} express the strength of the electron-hole interaction, while the non-diagonal components correspond to the exciton-biexciton interaction strength. The proportional to H_{ij} nonlinear terms describe effects of exciton-exciton interaction. In generally, the four-particle part of the equations should include contributions of pair-pair correlation functions and biexcitons. However, when the excitations fields are not too strong, the first quantity can be neglected (see, e.g., Ref. [7], text after eq.(13)), and our equations include only the biexciton terms.

The exciton-exciton interactions are described by matrix elements:

$$\mathrm{H}_{\mathrm{ii}\mathbf{kqpQ}}(\mathrm{t},\mathrm{t}') = \frac{1}{3!}\iint\iint \mathrm{d}\mathrm{r}\,\mathrm{d}\mathrm{r}'\,\mathrm{d}\mathrm{r}''\,\mathrm{d}\mathrm{r}''' \,\varphi_{\mathbf{k}}^{c_i*}(\mathrm{r})\varphi_{\mathbf{k}}^{\mathrm{v_i}}(\mathrm{r})\varphi_{\mathbf{q}}^{c_i*}(\mathrm{r}')\varphi_{\mathbf{q}}^{\mathrm{v_i}}(\mathrm{r}')\mathrm{h}_{\mathrm{XC}}(\mathrm{r},\mathrm{t};\mathrm{r}',\mathrm{t};\mathrm{r}'',\mathrm{t}';\mathrm{r}''',\mathrm{t}')\varphi_{\mathrm{p}}^{c_i}(\mathrm{r}'')\varphi_{\mathrm{p}}^{\mathrm{v_i}*}(\mathrm{r}'')\varphi_{\mathrm{Q}}^{c_i}(\mathrm{r}''')\varphi_{\mathrm{Q}}^{\mathrm{v_i}*}(\mathrm{r}''') \qquad (\mathrm{B}.10)$$

and

$$
\mathrm{H_{ij\mathbf{kqpQ}}}(t,t') = \frac{1}{3!}\iint\iint drdr'dr''dr''' \varphi_{\mathbf{k}}^{c_i*}(r)\varphi_{\mathbf{k}}^{v_i}(r)\varphi_{\mathbf{q}}^{c_j*}(r')\varphi_{\mathbf{q}}^{v_j}(r')\, h_{XC}(r,t;r',t;r'',t';r''',t')\Big[\varphi_{p}^{c_i}(r'')\varphi_{p}^{v_i*}(r'')\varphi_{Q}^{c_j}(r''')\varphi_{Q}^{v_j*}(r''') + \varphi_{Q}^{c_i}(r'')\varphi_{Q}^{v_i*}(r'')\varphi_{p}^{c_j}(r''')\varphi_{p}^{v_j*}(r''')\Big]. \qquad (B.11)
$$

The last lines in Eqs. B7-B8 give the biexciton contribution to the third-order polarization. Like excitons, one can expand the wave function in terms of product of two static wave functions

$$
\Psi_{k_1k_2}^{v}(r_1,r_2,t) = \sum_{l=v,c} D_{k_1k_2}^{lm}(t)\psi_{k_1}^{l}(r_1)\psi_{k_2}^{m}(r_2), \qquad (B.12)
$$

and write down the equation of motion for $D_{k_1k_2}^{lm}(t)$. Next, one can construct the density matrix for the two-particle states

$$
\Gamma_{k_1k_2k_3k_4}^{ablm}(t) = D_{k_1k_2}^{ab}(t)C_{k_3}^{l*}(t)C_{k_4}^{d*}(t) \qquad (B.13)
$$

and write down the equation of motion for this function, by using the equations for $C_k^{l*}(t)$, $-\mathrm{i}\frac{\partial \mathrm{C}_{\mathrm{k}}^{l*}(\mathrm{t})}{\partial \mathrm{t}} = \sum_{m=v,c} \mathrm{C}_{\mathrm{k}}^{\mathrm{m}*}(\mathrm{t})H_{kk}^{ml}(t)$ (see the case of excitons) and $D_{k_1k_2}^{ab}(t)$. Finally, the equation for the component $\Gamma_{k_1k_2k_3k_4}^{ccvv}(t) \equiv \mathrm{B}_{k_1k_2k_3k_4}^{ablm}(t)$ satisfying the equations

$$
\begin{aligned}
i\frac{\partial B_{k_1k_2k_1k_2}^{c_ic_iv_jv_j}(t)}{\partial t}
&= \left(\varepsilon_{k_1}^{c_i} + \varepsilon_{k_2}^{c_j} - \varepsilon_{k_1}^{v_i} - \varepsilon_{k_2}^{v_i} - \frac{i}{\tau_{XXij}}\right)B_{k_1k_2k_1k_2}^{c_1c_2v_1v_2}(t) + \sum_{q} F_{k_1k_1qq}B_{qk_2k_1k_2}^{c_ic_iv_jv_j}(t) \\
&+ \sum_{q} F_{k_1k_2qq}B_{qk_2k_1k_2}^{c_ic_iv_jv_j}(t) + \sum_{q} F_{k_2k_1qq}B_{k_1qk_1k_2}^{c_ic_iv_jv_j}(t) + \sum_{q} F_{k_2k_2qq}(t)B_{k_1qk_1k_2}^{c_ic_iv_jv_j}(t) \\
&+ \sum_{p,q} w_{k_1k_2pq}^{cccc}B_{pqk_1k_2}^{c_ic_iv_jv_j}(t) + \sum_{p,q} w_{k_1k_2pq}^{vvvv}B_{k_1k_2k_1k_2}^{c_ic_iv_jv_j}(t) \\
&+2F_{k_2k_2k_1k_1}\mathrm{P}_{\mathrm{i}\mathbf{k}_1}(\mathrm{t})\mathrm{P}_{\mathrm{j}\mathbf{k}_2}(\mathrm{t}) + F_{k_1k_1k_2k_2}\mathrm{P}_{\mathrm{i}\mathbf{k}_2}(\mathrm{t})\mathrm{P}_{\mathrm{j}\mathbf{k}_2}(\mathrm{t}) + 2F_{k_2k_2k_2k_2}(t)\mathrm{P}_{\mathrm{i}\mathbf{k}_2}(\mathrm{t})\mathrm{P}_{\mathrm{j}\mathbf{k}_1}(\mathrm{t}) + F_{k_2k_2k_1k_1}(t)\mathrm{P}_{\mathrm{i}\mathbf{k}_1}(\mathrm{t})\mathrm{P}_{\mathrm{j}\mathbf{k}_1}(\mathrm{t}), \qquad (B.14)
\end{aligned}
$$

where we have dropped the band indices for the F and w matrix elements. The last three lines in Eq. (B.14) are the source terms.

To solve the TD-DMDFT equations, Eqs. B7-B8 and B14, we used the screened Slater XC potential with the adiabatic XC kernels $f_{XC}^{Slater}(rt;r't')$

$$f_{XC}^{Slater}(rt;r't') = -\frac{2\left|\sum_{j,k}\psi_k^j(r)\psi_k^{j*}(r')\right|^2}{\varepsilon|r-r'|n_0(r)n_0(r')}\delta(t-t'), \qquad (B.15)$$

where $\varepsilon = 3.6$ is the static dielectric function, and $n_0(r)$ is the ground-state charge density,

and

$$h_{XCij}(r,t;r',t';r'',t'';r''',t''') = \delta(r-r')\delta(r'-r'')\delta(r'-r'')\delta(t-t')\delta(t''-t''')$$
$$\times \frac{\delta^2 f_{XC}^{Slater}(rt;r't')}{\delta n(r''t'')\delta n(r'''t''')} j_{XCij}(t-t''). \qquad (B.16)$$

In Eq. (B.16), the nonadiabatic part is (in frequency representation):

$$j_{XCij}(\omega) = \frac{1}{\omega - E_{XXij} + \frac{i}{\tau_{XXij}}}, \qquad (B.17)$$

where E_{XXij} and τ_{XXij} are the biexciton energy and lifetime correspondingly. The first quantities were calculated by solving the (exciton and biexciton) static eigen-problems and τ_{XXij} were chosen to fit the exp. data (see below).

In this paper, we apply Eqs. B7-B8 and B14 into a four-band problem to describe the dynamics of the two types of excitons formed at the K and K' valleys of the BZ, and the intra-valley biexciton. The first step of the calculation is to solve the eigenvalue equations for the excitons and biexcitons which are obtained by keeping the linear-in-charge terms in the r.h.s. of Eqs. B7-B8 and B14 and calculate, in this way, the exitonic and biexciton binding energies. The resulting energies, $E_{X1} = E_{X2} = 0.388\, eV$ and $E_{XX12} = E_{XX21} = 0.029\, eV$, are in a good agreement with experimental estimations (500-550meV for excitons [8-11] and 20meV for biexcitons [12]. Afterwards, the full non-linear equations of motion are solved within the excitonic basis. In this way, Eqs. B7-B8 and B14 transform to

$$\left(i\frac{\partial}{\partial t} - E_{X1} + \frac{i}{\tau_{X1}}\right) P_1(t)$$
$$= \boldsymbol{d_1 E}(t) + 3P_1^*(t) \int_{-\infty}^{t} dt' \, H_{11}(t, t') P_1(t') P_1(t')$$
$$+ 9P_2^*(t) \int_{-\infty}^{t} dt' \, H_{12}(t, t') P_1(t') P_2(t')$$
$$+ 9\left(F_{21}^* P_1^*(t) + F_{22}^* P_2^*(t)\right) B_{12}(t), \qquad (B.18)$$

$$\left(i\frac{\partial}{\partial t} - E_{X2} + \frac{i}{\tau_{X1}}\right) P_2(t)$$
$$= \boldsymbol{d_2 E}(t) + 3P_2^*(t) \int_{-\infty}^{t} dt' \, H_{22}(t, t') P_2(t') P_2(t')$$
$$+ 9P_1^*(t) \int_{-\infty}^{t} dt' \, H_{21}(t, t') P_2(t') P_1(t')$$
$$+ 9\left(F_{12}^* P_2^*(t) + F_{11}^* P_1^*(t)\right) B_{21}(t), \qquad (B.19)$$

$$\left(i\frac{\partial}{\partial t} - \mathrm{E}_{12}^{\mathrm{XX}} + \frac{i}{\tau_{XX12}}\right) B_{12}(t) = 3F_{21}P_1(t)P_1(t) + 6F_{11}P_1(t)P_2(t) + 6F_{22}P_2(t)P_1(t) + 3F_{12}P_2(t)P_2(t). \qquad (B.20)$$

$$\left(i\frac{\partial}{\partial t} - \mathrm{E}_{21}^{\mathrm{XX}} + \frac{i}{\tau_{XX21}}\right) B_{21}(t) = 3F_{12}P_2(t)P_2(t) + 6F_{22}P_2(t)P_1(t) + 6F_{11}P_1(t)P_2(t) + 3F_{21}P_1(t)P_1(t). \qquad (B.21)$$

In the last equations E_{Xi} and E_{XXij} are the energy of i's exciton, and of the $ij's$ biexciton, and τ_{Xi} and τ_{XXij} are the corresponding lifetimes ($i, j = 1,2$). d_i is the dipole moment for the transition in i's valley, and

$$\mathrm{H_{ij}}(\mathrm{t}, \mathrm{t}') = \frac{1}{3}\int dr \left|\psi_{\mathrm{K}_i}^{c_i}(\mathrm{r})\psi_{\mathrm{K}_i}^{\mathrm{v_i}*}(\mathrm{r})\right|^2 \mathrm{h_{XC}}(\mathrm{r}) \left|\psi_{\mathrm{K}_j}^{\mathrm{c}_j}(\mathrm{r})\psi_{K_j}^{\mathrm{v_j}*}(\mathrm{r})\right|^2 e^{-i\left(E_{XXij} - \frac{\mathrm{i}}{\tau_{\mathrm{XXij}}}\right)(\mathrm{t}-\mathrm{t}')}, \qquad (B.22)$$

where the kernel

$$\mathrm{h_{XC}}(\mathrm{r}) = \delta(\mathrm{r} - \mathrm{r}')\delta(\mathrm{r} - \mathrm{r}'')\delta(\mathrm{r} - \mathrm{r}''')\frac{\delta^2 \mathrm{f}_{\mathrm{XC}}^{\mathrm{Slater}}(\mathrm{r}; \mathrm{r}')}{\delta \mathrm{n}(\mathrm{r}'')\delta \mathrm{n}(\mathrm{r}''')}$$

is obtained by performing functional differentiation of the Slater XC kernel.

For the excitonic decay times τ_X we chose 170 fs (B=0T, co-circular), 228 fs (B=0T, cross-circular), 184 fs (B=25 T, co-circular) and 359 fs (B=25 T, cross-circular). To be consistent in the choice of the excitonic and biexciton decay times, we have used the biexciton lifetime τ_{XX} two times smaller than the corresponding excitonic times above. The zero-field excitonic lifetimes are in a good agreement with other estimations (~240 fs at zero field in the zero-temperature limit, see e.g., [15]) and experimental results [16]. To mimic the effect of the magnetic field that reduces the number of the excited carriers, and hence the screening (which results in longer lifetimes), we have

used longer lifetimes in the case of magnetic field. Indeed, as it was shown for bulk GaAs, decreasing of carrier doping in a presence of a magnetic field can reduce the excitonic rise and decay time parameters by several times (see Fig.22 in Ref. [7]). Finally, we have used longer biexciton (and hence excitonic) lifetimes in the case of cross-circular pulses, since in this case excitons attract stronger. It must be noted that in the zero-field case the difference between the used values of the co- and cross- lifetimes is not very large. Indeed, in zero field the screening effects are not dominating, thus they should not result in a significant difference in the attraction between the exciton for different pulse polarizations.

The non-linear equations of motion are then solved in the excitonic basis and the TI-FWM spectrum is given by

$$\mathrm{I}(\tau, \mathrm{T}=0)=\int_{0}^{\infty}\left|\mathrm{P}^{(3)}(\tau, \mathrm{T}=0)\right|^{2} \mathrm{dt}, \qquad (B.23)$$

where $P^{(3)}(\tau, T=0)$ is the sum of all $P_1(t)$ and $P_2(t)$ obtained by solving Eqs B19-21 assuming the two different polarization sequences used in the TI-FWM experiments.

Non-Markovian memory effects and the biexciton decay

Here we discuss the decay dynamics of the biexciton, where we follow the approach introduced in Ref. 17 to describe non-Markovian memory effects in bulk GaAs under magnetic fields.

Let's consider a formal solution for the biexcitonic matrix element B(t) (Eqs. (B.20-21), $B(t)\sim\int_{-\infty}^{t} P^2(t') e^{\left(iE^{xx}-\frac{1}{\tau_{XX}}\right)(t-t')}$. As it follows from this expression, in the limit of very long times, $B(t)$ is exponentially decaying (the decay is defined by the factor $\frac{1}{\tau_{XX}}$), and at short times $B(t)$ grows as square of polarization. In the intermediate case of finite but not short times the corresponding "polarization-correction" to the exponential decay of $B(t)$ can be important for a rather long time interval causing some quantitative slowing down of the biexciton rate.

It is the interplay between the polarization square and exponential decay terms, a consequence of non-Markovian memory effects, which lead to decay times for the biexciton that far exceed τ_{XX}. For a more in-depth discussion please refer to Refs. 17-18.

Supplementary note 3: Sample and experiment

Sample: The atomic monolayer sample was grown using a chemical-vapor-deposition (CVD) system, synthesized on Si substrates with a 300 nm SiO_2 layer at 800±10 °C directly from $MoSe_2$ bulk powder used as a solid precursor and N_2+H_2O vapor as carrier gas (details are provided in Ref. 19). The monolayer $MoSe_2$ samples (5x5mm area) were subsequently wet-transferred using PMMA to double side polished quartz-substrate.

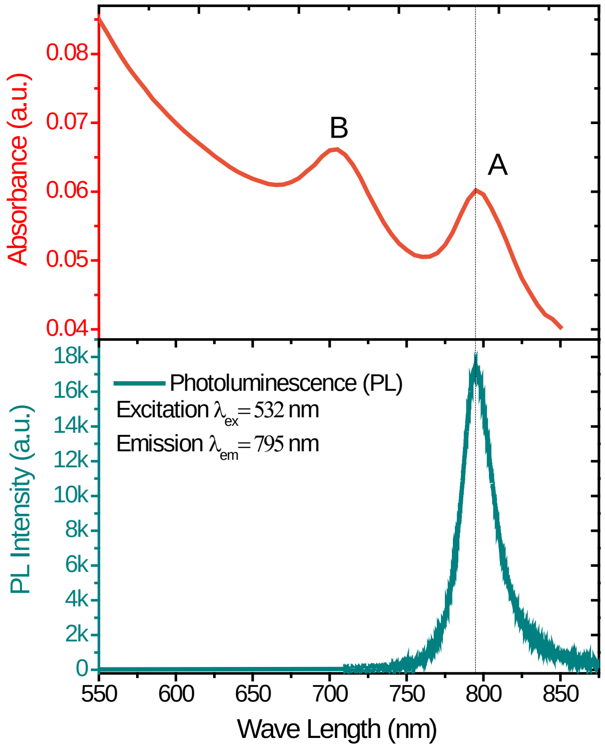

Supplementary Figure 4: The optical absorption measurements of monolayer $MoSe_2$ grown directly on quartz reveal two strong excitonic features near 795 nm, and 705 nm corresponding to the A, and B excitons respectively. The nearly 90 nm (~200 meV) splitting of the A and B excitons is consistent with earlier reports (Ref. 19-20). In addition, the μ-PL spectra of the similar $MoSe_2$ monolayer sample on Quartz substrate show exactly a strong peak only at 795 nm. This peak position can be attributed to the A exciton in $MoSe_2$ monolayer. Interestingly, the A exciton peak position matches very well in the two experiment.

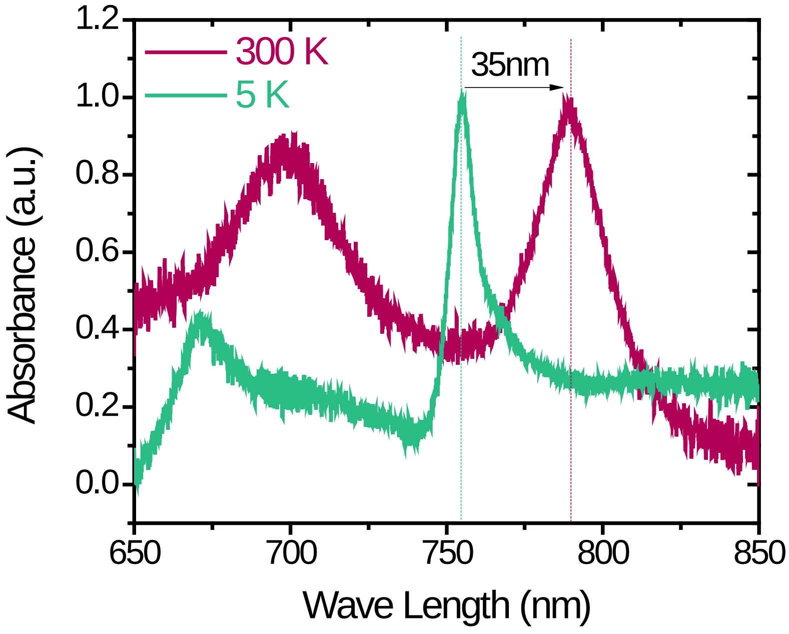

Supplementary Figure 5: The figure shows the absorption spectra recorded from a mechanically exfoliated $MoSe_2$ sample at two different temperature; Green spectrum at 5K and Red spectrum at 300K. These result clearly indicates that the A exciton band at 5K is redshifted by ~35 nm upon increasing the temperature to 300K.

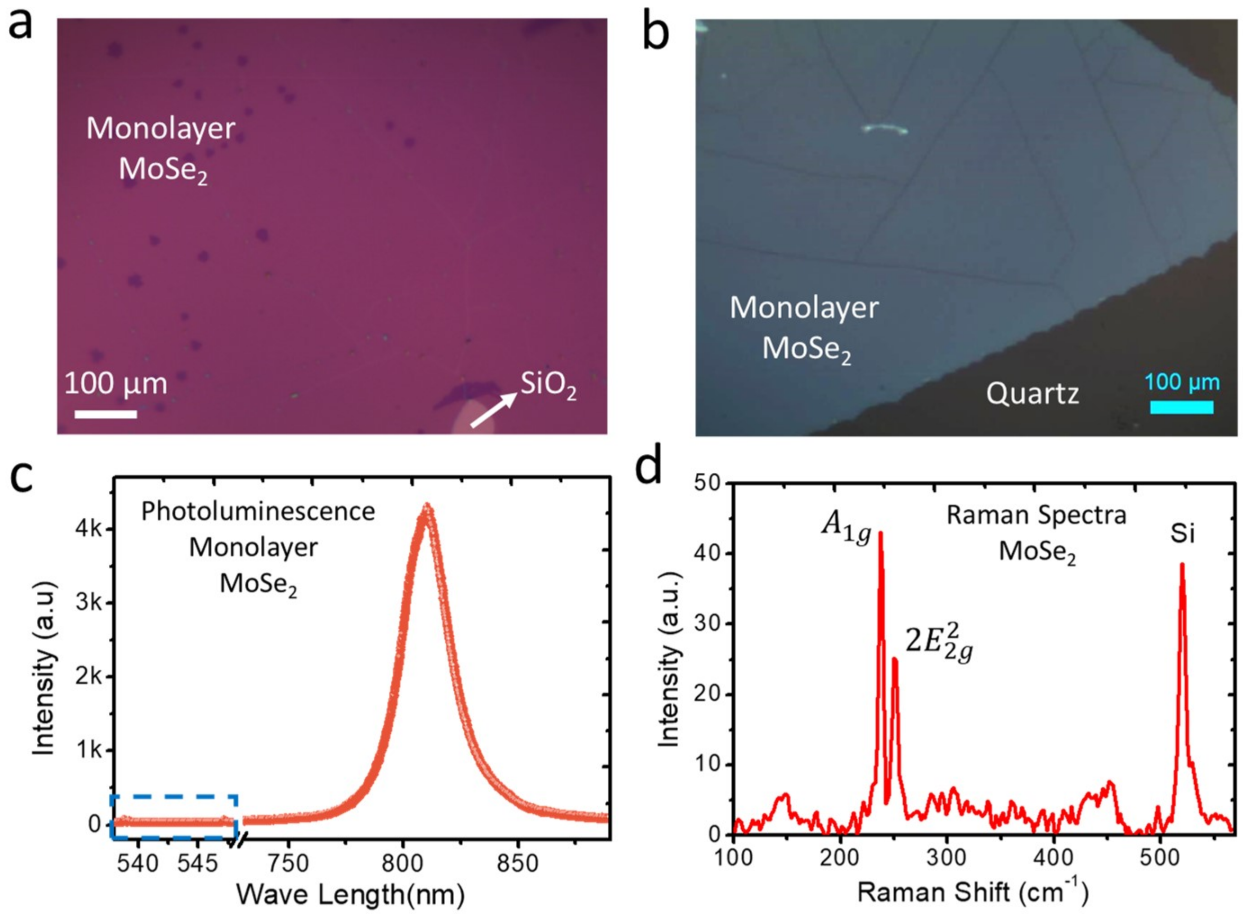

Supplementary Figure 6: Optical microscopy image of **(a)** monolayer $MoSe_2$ directly synthesized on 300 nm thick SiO_2 layer on Si substrate, and **(b)** monolayer $MoSe_2$ directly synthesized on quartz substrate via chemical vapor deposition process. **(c)** photoluminescence (PL) spectra corresponds to monolayer $MoSe_2$. The µ-PL spectra show a strong peak only around 810 nm, attributed to the direct excitonic transition energy in monolayer $MoSe_2$. **(d)** Raman spectrum of monolayer $MoSe_2$.The most dominant Raman peak at 240 cm^{-1} is belongs to A_{1g} mode of monolayer $MoSe_2$ [21]. The additional shoulder at 249 cm^{-1} can be assigned to the E_{2g}^2 shear mode of $MoSe_2$ at the M point. The high PL/Raman peak ratio (indicated by dashed box in (c)) indicates that the as-grown $MoSe_2$ monolayer are high crystalline in nature.

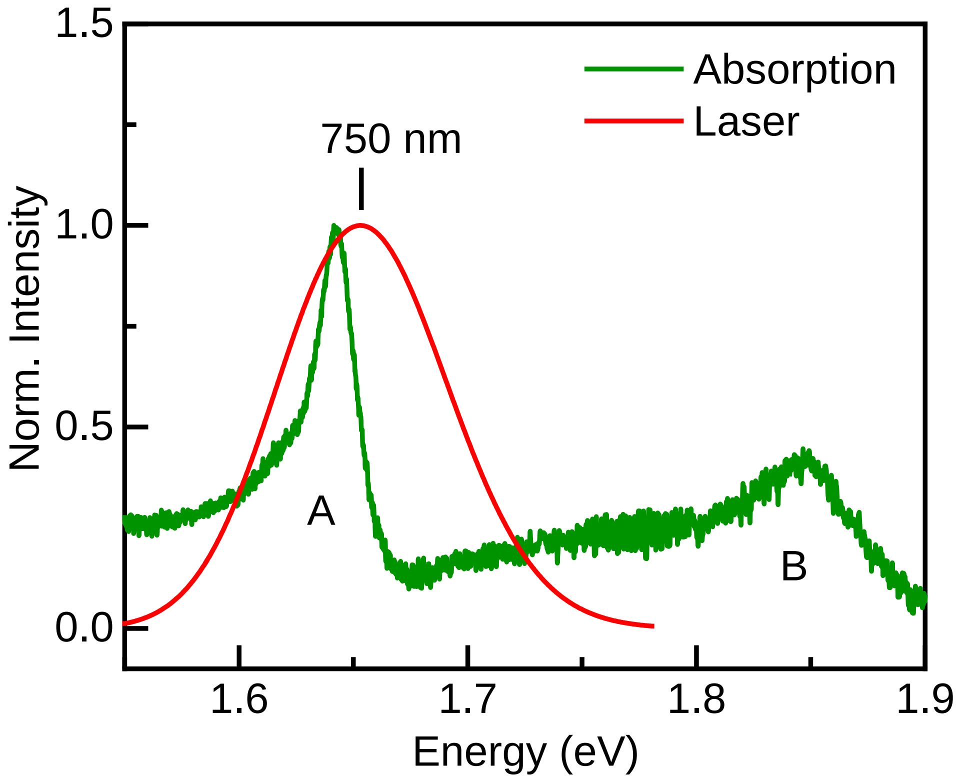

Supplementary Figure 7: (Green line) Absorption spectra of monoatomic $MoSe_2$ at ~10 Kelvin. The spectra are normalized to the A exciton. Both A and B excitons can be observed. (Red line) The excitation laser spectrum centered ~750 nm.

Experiment: The three laser beams used to generate the FWM signal are provided by the multidimensional optical nonlinear spectrometer (MONSTR) instrument described in Ref. 22. Pulses B and C are kept fixed whereas pulse A* is scanned from negative to positive time delays. The positive delay signal corresponds to the time ordering of the laser pulses shown in Fig. S8 (c), whereas the negative delay signal time ordering shown in Fig. S8 (d). The nonlinear third order response of the sample leads to the FWM signal, which propagates in the phase matching direction $-\mathbf{k}_1 + \mathbf{k}_2 + \mathbf{k}_3$ along the missing corner of the box formed by the three excitation laser pulses, as shown in Fig. S8 (a), and the coherent time-integrated FWM signal is collected by the detector. The monolayer $MoSe_2$ sample is held at 10 Kelvin inside the resistive 25 Tesla split helix magnet. The magnetic field and laser excitations are applied perpendicular to the sample surface shown in Fig. S5 (b).

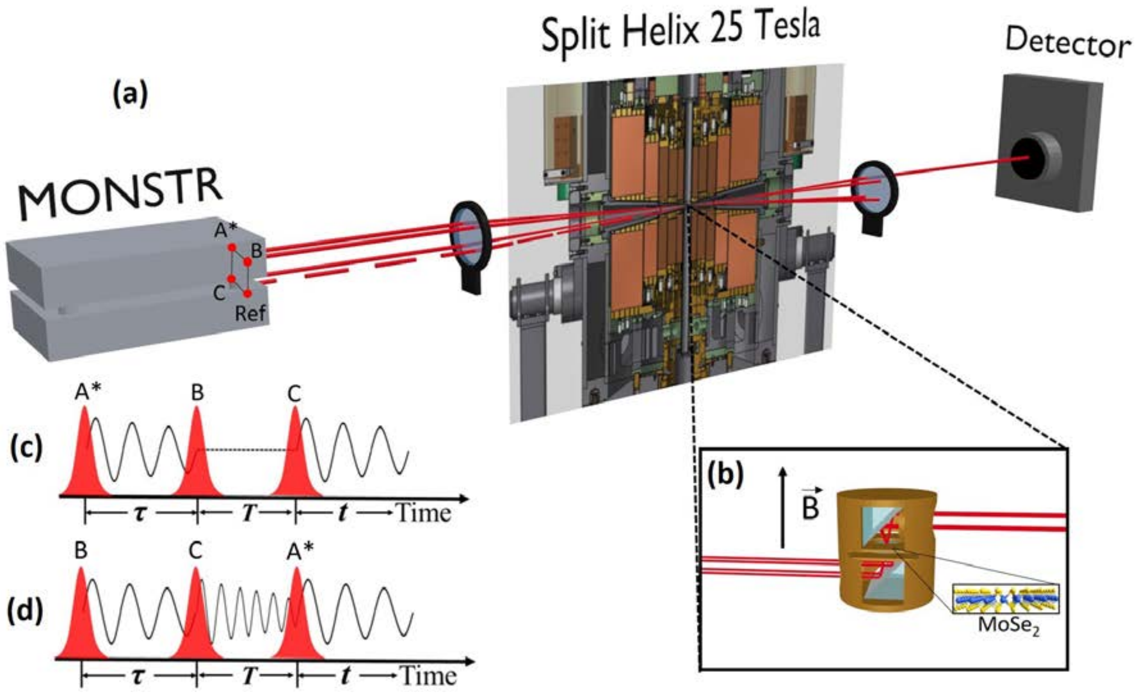

Supplementary Figure 8: (a) Schematic of the experimental setup: The three laser beams are provided by the MONSTR [22]. Three beams labeled as A*, B, and C are used to generate the FWM signal, where A* corresponds to the phase conjugate beam. The beams are aligned in the three corners of a square. The FWM signal generated at the sample propagates along the missing corner (direction $-\mathbf{k}_1 + \mathbf{k}_2 + \mathbf{k}_3$) and is collected by the detector. The samples are kept at 10 Kelvin inside the magneto-optical cryostat. (b) The magnetic fields up to 25 Tesla are applied perpendicular to the sample surface in Faraday geometry. (c) Pulse sequence leading to positive delay FWM signal. (d) When the phase conjugate pulse A* arrives last, the negative delay signal is generated due to multiple exciton correlations.

The laser pulses with a temporal linewidth ~76.5 fs are plotted in the figures as the instrumental response. The spectral linewidth was measured by dispersing the laser pulse using a grating spectrometer and corresponded to 39.6 ± 2.3 nm full-width at half maximum. The excitation power used was 300 μW average power centered at ~750 nm. Measuring a spot size of ~500 microns, we obtain a laser fluence of ~4 x 10^{-9} J/cm^2 and power density of ~9.55 x 10^{-3} W / cm^2.

Supplementary References:

[1] P. Giannozzi *et al*., J. Phys.: Condens. Matter **21,** 395502 (2009).

[2] Y. Zhang *et al*., Nature Nanotech. **9** ,111 (2014).

[3] Z. Wang *et al*., Nano Lett. **17**, 740 (2017).

[4] Xiao D. *et al*., PRL **108**, 196802 (2012).

[5] Li Y. *et al*., PRL **110**, 066803 (2013).

[6] V. Turkowski and M.N. Leuenberger, Phys. Rev. B **89**, 075309 (2014).

[7] Kner, Bar-Ad, Marquezini, Chemla, Lovenich, Schaefer *et al*., PRB **60**, 4731 (1999).

[8] J. S. Ross *et al*., Nat. Commun. **4**, 1474 (2013).

[9] Y. Li *et al*., PRL **113**, 266804 (2014).

[10] Wang *et al*., 2D Mater. **2**, 045005 (2015).

[11] A. Arora *et al*., Nanoscale **7**, 20769 (2015).

[12] K. Hao *et al*., Nature Commun. **8**, 15552 (2017).

[13] Zhang *et al*., Nature Nanotech. **9** ,111 (2014).

[14] Wang *et al*., Nano Lett., **17**, 740 (2017)**.**

[15] Palummo *et al*., Nano Lett. **15**, 2794−2800 (2015).

[16] Moody *et al.*, Nature Commun. **6**, 8315 (2015)

[17] J. Shah, "Ultrafast properties of Semiconductors and Semiconducting Nanoparticles" (Springer, 1998).

[18] P. Kner et al., Phys. Rev. B 60, 4731 (1999).

[19] Y. Li et al. Phys. Rev. B: Condens. Matter Mater. Phys. 2014, 90, 205422,

[20] Kirby K. H. Smith et al. ACS Appl. Nano Mater., 2018, 1, 572–579).

[21] P. K. Sahoo, *et al*., Nature **553**, 63 (2018).

[22] P. Dey, J. Paul, J. Bylsma, S. Deminico, D. Karaiskaj, Rev. Sci. Instrum. **84**, 023107 (2013).
